# A case report of a deep surgical site infection with *Terrisporobacter glycolicus/T. Mayombei* and review of the literature

**DOI:** 10.1186/s12879-016-1865-8

**Published:** 2016-09-29

**Authors:** Matthew P. Cheng, Marc-Christian Domingo, Simon Lévesque, Cedric P. Yansouni

**Affiliations:** 1 Division of Infectious Diseases and Department of Medical Microbiology, Glen site, McGill University Health Centre, 1001 Boulevard Décarie, Room E05. 1811.2, Montreal, Quebec H4A 3J1 Canada; 2 Laboratoire de santé publique du Québec, McGill University Health Centre, Montreal, Canada; 3 J.D. MacLean Centre for Tropical Diseases, McGill University Health Centre, Montreal, Canada

**Keywords:** Surgical site infection, *Clostridium glycolicum*, *Terrisporobacter glycolicus*, *Terrisporobacter mayombei*, MALDI-TOF, PCR

## Abstract

**Background:**

There are increasing data regarding *Terrisporobacter glycolicus* as an emerging anaerobic pathogen. However, the few published cases to date usually report it as part of a polymicrobial infection. Here, we describe the first reported monomicrobial surgical site infection with this bacterium. Identification methods, taxonomy, and clinical management of this rarely identified pathogen are also discussed.

**Case presentation:**

A previously healthy 66-year-old sustained an open olecranon fracture of his left arm after trauma. He subsequently underwent open reduction and internal fixation (ORIF), with insertion of an olecranon locking plate and two locking screws. Ten days after surgery, the patient developed increasing pain at the surgical site and noted green discharge from the wound. Culture of the wound discharge yielded grew a pure Gram-positive anaerobe identified by the RapidANA® microbial identification system as *C. difficile* (profile 000010, 99.1 % probability). Reference laboratory testing identified the isolate as *T. glycolicus/mayombei (previously designated as Clostridium glycolicum/mayombei)* by 16S rRNA gene sequencing and as *Clostridium glycolicum by* MALDI-TOF mass spectrometry. The patient received an 8-week course of moxifloxacin and metronidazole with an excellent clinical response at 12 months’ follow-up.

**Conclusions:**

We describe the case of a deep surgical site infection with *T. glycolicus/mayombei* (formerly known as *Clostridium glycolicum* and *Clostridium mayombei*, respectively), which extends our knowledge of the clinical spectrum of *this pathogen*. The isolate was misidentified by phenotypic identification methods.

## Background

*Clostridium* spp*.* are a heterogeneous group of anaerobic, spore-forming, gram-positive rod-shaped bacteria. Over 200 distinct species have been identified, most of which are environmental organisms; few species have been recognized to cause clinical disease [1]. There are increasing data regarding *Terrisporobacter glycolicus & Terrisporobacter mayombei* as emerging anaerobic pathogens, yet relatively little is known about their pathogenic potential. Previous case reports of *C. glycolicum* (the former designation of *T. glycolicus*) identified the bacteria in cultures of wounds and blood, often in conjunction with other pathogens [2–4]. Although it has been implicated in bloodstream infections, brain abscesses as well as bone and joint infections, its ideal method of identification has yet to be determined.

We describe the first reported monomicrobial surgical site infection with prosthetic material from * T. glycolicus*/*T. mayombei*. Phenotypic identification systems used in our laboratory at the time of the case identified it as *C. difficile,* but 16S ribosomal sequence analysis yielded *T. glycolicus/T. mayombei* with a high degree of certainty. In addition, MALDI-TOF MS system demonstrated the ability to correctly identify *C. glycolicum*, under its former designation.

## Case presentation

A previously healthy 66-year-old man fell off of his bicycle onto dry pavement, and sustained an open olecranon fracture of his left arm. He subsequently underwent open reduction and internal fixation (ORIF), with insertion of an olecranon locking plate and two locking screws. Chlorhexidine-based sterilizing solution and peri-operative cefazolin were used in accordance with our institution’s protocols. The procedure was uncomplicated and the patient was discharged home on the third post-operative day.

Ten days after surgery, the patient developed increasing pain at the surgical site and noted green discharge from the wound. He was re-admitted to hospital for surgical drainage of the affected elbow, and piperacillin-tazobactam was prescribed empirically. Culture of the wound discharge yielded anaerobic spore-forming Gram-positive rods in broth media and anaerobic 5 % sheep blood agar, identified by the RapidANA® microbial identification system as *C. difficile* (profile 000010, 99.1 % probability). The aerobic cultures were negative. Given the rarity of *C. difficile* joint infections [5, 6], was reported as “*Clostridium* species” and the isolate was sent to the provincial reference laboratory for speciation. Our patient did not have any evidence of *C. difficile*-associated diarrhea and his stool specimen was negative for *C. difficile* toxin by PCR. He was subsequently treated with intravenous vancomycin and oral metronidazole, pending results of antimicrobial susceptibility testing.

At the Laboratoire de santé publique du Québec (LSPQ), latex agglutination (Microgen Bioproducts Ltd) yielded a positive result indicating a possible *C. difficile* strain. However, according to the manufacturer package insert, *C. glycolicum* is known to cross react with this reagent. The isolate was identified as *C. glycolicum* by MALDI-TOF mass spectrometry using the bioMérieux VITEK MS system (IVD database version 2.0). The clinical isolate, designated LSPQ-04251, was definitively identified by 16S rRNA gene sequencing using a BigDye method with ABI3130 XL sequencing (Applied Biosystems, Foster City, CA). Pairwise nucleotide sequence similarities of the 16S rRNA gene indicated that the closest known relatives of the isolate were *Clostridium glycolicum* (99.3 %) (recently reclassified as *Terrisporobacter glycolicus*), and *Clostridium mayombei* (99.2 %) (recently reclassified as *Terrisporobacter mayombei*) [7]. Based on the 16S rRNA gene sequence comparison, *T. glycolicus* and *T. mayombei* were genetically highly related, displaying 99.4 % sequence similarities with 0.6 % divergence. No further characterization of isolate LSP-04251 to separate both species *T. glycolicus* and *T. mayombei*, (requiring additional analysis of the genomic DNA G + C content, predominant cellular fatty acids and products from peptone yeast extract) was performed [7].

As there is little experience with clinical infections from these bacteria [8], we relied on minimal inhibitory concentration (MIC) determination by Etest®, according to the manufacturer’s application guide, to guide antimicrobial treatment (see Table 1). Treatment was changed to oral moxifloxacin and metronidazole due to logistical barriers to intravenous therapy in this patient and their excellent bioavailability and bone penetration [9, 10], for a total of 8 weeks. One year after stopping antibiotics, the patient was well, without signs of recrudescent infection.

**Table 1:** Minimal inhibitory concentration (MIC; μg/mL) of the clinical isolate of *T. glycolicus/T. mayombei,* as determined by Etest®

Antimicrobial	MIC (μg/mL)
Piperacillin/Tazobactam	1.5
Penicillin	1
Metronidazole	0.5
Meropenem	1
Clindamycin	0.125
Cefoxitin	32
Ceftriaxone	32
Imipenem	32
Moxifloxacin	1

## Discussion

Surgical site infections are an important complication of open fractures, and this risk is modulated by several variables including the size of the wound, the complexity of the fracture, and the presence of necrotic soft tissue [11]. Our patient’s wound was classified as Gustilo Type II (i.e. open fracture with a laceration <1 cm in length without extensive soft tissue damage, flaps, or avulsions) without severe contamination. No antimicrobial prophylaxis was given beyond the surgical procedure. It is most plausible that our patient’s wound was infected with *T. glycolicum*/*T. mayombei* at the time of his initial injury on the pavement. Although we cannot exclude haematogenous seeding from the gastrointestinal tract, this would seem unlikely given the circumstances of the accident, the organism identified and the absence of GI symptoms or underlying disease.

Although not currently assigned formally to the family *Peptostreptococcaceae*, phylogenetic analyses indicated that the species *Terrisporobacter glycolicus* and *T. mayombei* are members of the family *Peptostreptococcaceae* [7]. To support this classification in the family of *Peptostreptococcaceae*, Yutin and Galpieri compared a phylogenetic tree for a concatenated set of 50 widespread ribosomal proteins with the trees for beta subunits of the RNA polymerase (RpoB) and DNA gyrase (GyrB) genes and with the 16S rRNA-based phylogeny tree. The result of this analysis support placement of *Clostridium difficile* and its close relatives (including species of the genus *Terrisporobacter*) within the family *Peptostreptococcaceae*. Hence, some have proposed to rename *C. difficile* as *Peptoclostridium difficile,* and to transfer *C. glycolicum* and *C. mayombei* to this genus as well [1]. This taxonomic controversy remains unresolved for the moment. Thus, based on available phylogenetic data, misidentification of the isolate LSPQ-04251 as *C. difficile* by phenotypic methods is not unexpected.

Relatively little is known about the pathogenic potential of *T. glycolicus*/*T. mayombei.* Elsayed and Zhang first reported a first case of human infection with *C. glycolicum* in 2007, when a 43 year old woman developed septic shock after a recent bone marrow transplant for relapsed Hodgkin’s disease [2]. Their patient was found to have a polymicrobial bactermia with *C. glycolicum* and *Enterococcus* spp*.* She received numerous antibiotics initially and subsequently completed a two-week course of ampicillin, gentamicin and metronidazole with a good clinical response. In 2009, Van Leer et al. described the case of a 62 year-old gentleman who developed a brain abscess with gas formation after otitis media, for which he treated himself with clay in his ear [4]. *C. glycolicum* was the only organism identified in the patient’s blood, but numerous other organisms were recovered from the pus and necrotic tissue from the mastoid. Their patient eventually recovered with residual bilateral hearing loss after surgical debridement of the infection. In 2012, Jiang et al. reported two cases of human infection of *C. glycolicum* after suffering accidental injuries. Both cases resulted in polymicrobial infections, with one patient developing a coagulase-negative staphylococcal bacteremia requiring six weeks of intravenous antibiotic therapy with vancomycin. Subsequently, Cai et al. described a case of a 67 year-old gentleman with numerous medical co-morbidities who developed acalculous cholecystitis and was found to have a bloodstream infection with *C. glycolicum.* No other pathogens were identified and the patient responded well to treatment with pipacillin-tazobactam.

## Conclusion

We describe the first reported monomicrobial surgical site infection from *T. glycolicus*/*T. mayombei*. Sequence analysis of the 16S rRNA gene offers greater discrimination between genera and, in many cases, species compared to the other bacterial identification methods described in this report. However, MALDI-TOF mass spectrometry also correctly identified *C. glycolicum* under its former designation and may be a reliable identification method for these pathogens. Our case demonstrates the limitations of phenotypic identification systems for clostridial species, and extends our knowledge of the clinical spectrum of *T. glycolicus*/*T. mayombei.* Finally, species identification for deep Gram-positive anaerobic rod infections is pertinent because antimicrobial susceptibility patterns may be poorly characterised or variable, and mandate susceptibility testing for rarely encountered species.

## Key points

- *Terrisporobacter glycolicus/Terrisporobacter mayombi* is a rare cause of clinical infection, but has been implicated in brain abscesses, bone and joint infections, as well as bloodstream infections.
- *T. glycolicus/mayombi* was misidentified as *C. difficile* by phenotypic and latex agglutination testing. Accurate identification was achieved with both 16S rRNA gene sequence analysis and MALDI-TOF MS.
- Identification to the species level for deep Gram-positive anaerobic rod infections is potentially clinically pertinent, and rarely encountered species should prompt antimicrobial susceptibility testing.

## Abbreviations

MALDI-TOF: Matrix-assisted laser desorption/ionization – time of flight
MIC: miminal inhibitory concentration
ORIF: Open reduction and internal fixation
